# Comprehensive Insight into the Male Dog Reproductive System in Health and Diseases: Developmental, Genetic, and Environmental Factors—Review on Research and Clinical Trials

**DOI:** 10.3390/vetsci13050464

**Published:** 2026-05-11

**Authors:** Aybike Turkmen, Grzegorz Lonc, Begum Yurdakok-Dikmen, Koray Tekin, Dorota Gil, Marta Zarzycka, Katarzyna Kania, Malgorzata Kotula-Balak

**Affiliations:** 1 Department of Animal Anatomy and Preclinical Sciences, Faculty of Veterinary Medicine, University of Agriculture in Krakow, Mickiewicza 24/28, 30-059 Krakow, Poland; grzegorz.lonc@urk.edu.pl (G.L.); karzyna.kania@student.urk.edu.pl (K.K.); 2 Department of Pharmacology and Toxicology, Faculty of Veterinary Medicine, Ankara University, 06070 Ankara, Turkey; yurdakok@veterinary.ankara.edu.tr; 3 Department of Reproduction and Artificial Insemination, Faculty of Veterinary Medicine, Ankara University, 06070 Ankara, Turkey; ktekin@ankara.edu.tr; 4 Chair of Medical Biochemistry, Jagiellonian University Medical College, Kopernika 7, 31-034 Krakow, Poland; dorotabeata.gil@uj.edu.pl (D.G.); marta.zarzycka@uj.edu.pl (M.Z.)

**Keywords:** male dog, reproductive system, sex hormones, infertility, testicular tumors, developmental disorders, environmental factors

## Abstract

As in other mammalian species, the complex and specific interactions between internal biological processes and external factors regulate and impact the male dog reproductive system functions. This comprehensive review integrates physiological and molecular mechanisms underlying the reproductive system maintenance throughout the anatomical and histological structure of reproductive organs and their functions from development to aging. Simultaneously, the presentation of fundamental hormonal regulations and functions of the reproductive system is comprised. Special attention is put on e.g., genetic, developmental, age- and environmental-related disorders. The structural and hormonal status of the reproductive organs in response to single or mixed influences: genetic predispositions (e.g., cryptorchidism, sex chromosome aneuploidy syndrome), developmental courses (e.g., cryptorchidism, *uterus masculinus*, hypospadias), age-related diseases (e.g., tumors), and environmental stressors: e.g., endocrine-disrupting chemicals, toxins, heat stress (possibly leading to e.g., hypogonadism, cryptorchidism, infertility, tumors, precocious aging) is provided. Such multidirectional and comprehensive associations of grouped, selected, clinically significant pathological processes and diseases are broadly considered and linked here for the first time. Based on both epidemiological and experimental findings, the etiologies, current diagnostic approaches, treatment options, and prognostic assessments of these common male dog disorders are presented. This compendium seems useful for young veterinarians, researchers, breeders, and dog owners, enabling them to integrate knowledge on biological principles and processes with clinical practices and research in recent and future canine andrology.

## Simple Summary

Currently, a better understanding of the male dog reproductive system in both health and disease is crucial not only for advancing healthcare approaches but also for raising awareness of preventable disruptive factors that can be avoided throughout the dog’s life. This review presents the development and physiology, including hormonal regulation of the male dog’s internal and external reproductive organs. However, our primary goal is to group and understand how genetic predispositions, development perturbations, aging, and environmental factors such as endocrine disrupters or heat stress alone and/or together impair canine reproductive health. We focus on the current literature data on selected but frequently occurring reproductive organ dysfunction, diagnosis, and treatment, with special attention to, e.g., genetic, developmental, age- and environment-related disorders. This compendium of knowledge can be useful not only for young veterinarians and scientists but also for dog breeders and dog owners.

## 1. Introduction

Reproduction in the domestic dog (*Canis familiaris*) is an important part of general breeding and veterinary practice in relation to both optimization of reproductive performance and its prevention [1]. In recent years, diagnostic and treatment approaches have advanced profoundly, leading to the successful management of many diseases. Current extensive research and epidemiological studies clearly demonstrate that environmental pollution significantly impairs reproductive health in both humans and animals [2]. Accompanying animals are affected by the same environmental factors (chemicals, lifestyle, heat stress) as their owners because they occupy the same place. In humans, infertility is increasing globally, and the male factor is responsible for more than 50% of failures. According to some recent studies, the dog is considered a sentinel species that shares similar health problems, including fertility disturbances [3].

The male reproductive system plays a complex role in sex hormone production (mainly in the testes), hormone secretion for local and peripheral regulations, production and partial maturation of spermatozoa in the testes, storage, full maturation and transport (epididymis and *vas deferens*), and deposition of spermatozoa in the female reproductive tract (penis). Therefore, its homeostasis needs to be constantly maintained *via* a healthy internal microenvironment and external influences (e.g., a healthy environment, a balanced diet). The main reproductive gland, the testis, is directly controlled by the central neuroendocrine hypothalamic–pituitary–gonadal axis (HPG) and local regulations supervising other reproductive organs [4,5]. These hormonal controls can be modulated by gene products of involved proteins and hormones, as well as negatively by external stressors (e.g., chemicals, heat stress, or nutrition) [5]. It is important to notice here that, present in the environment, hormonally active chemicals (endocrine disrupters), due to their estrogenic or antiandrogenic nature, affect the sex hormone signaling through intracellular androgen and estrogen receptors (AR and ER), which modulate the expression of dependent genes [6,7]. Additionally, newly discovered receptors located on the cell membrane are involved in this process [8,9], triggering fast cellular responses such as proliferation and apoptosis. The disruption of hormonal transduction mechanisms has negative and often transgenerational effects on the developmental, genetic, and functional status of the reproductive system [10]. Spermatozoa, like other cells of the reproductive organs, express sex hormone receptors, which are a target for endocrine disruptors. This was confirmed in large studies of the stud dog population in which sperm motility and viability declined over 26 years, and chemicals were detected in the testis and ejaculate [11]. In recent years, the presence of significant concentrations of various chemicals in human and animal semen, breast milk, and placenta has been frequently reported [12,13] together with alterations in anogenital distance in newborns that indicate hormonal imbalances during the developmental period [14] have been frequently reported. Moreover, some therapeutics used in animal clinical procedures may concomitantly affect the function of the reproductive system [15]. In dogs, the genetic predispositions (breed-specific, related to selective breeding, or age) are an important group of factors negatively affecting the reproductive system [16]. Some systemic disorders with a genetic basis, e.g., canine sex chromosome aneuploidy syndrome or cryptorchidism, are highly related to the risk of tumor development [17,18]. Besides the above, other diverse conditions disturb proper reproductive system function *via* changes in specific microenvironment or by causing perturbations in protective cellular and molecular barriers (e.g., sexually transmitted infectious diseases, surgical or traumatic injuries, and intensive use for reproduction) [1,18]. In light of the above facts, this review may serve as an additional comprehensive information source, especially for not fully experienced clinical practitioners, researchers, breeders, or owners. It may direct further searches for new and linked data on the male dog reproductive organs, from microanatomy and histology to molecular and environmental mechanisms, in both health and disease conditions.

## 2. Male Dog Reproductive System Development

Despite progress in veterinary science, several aspects of the embryology and development of the male dog reproductive system remain not fully known. Some main developmental events are presented here, referring to general knowledge in mammals. In early mammalian embryonic development, the bipotential gonad, a pair of longitudinal ridges derived from intermediate mesoderm, and the overlying epithelium are first formed. Next, germ cells begin to migrate from the endoderm lining of the yolk sac to the genital ridges, *via* the dorsal mesentery of the hindgut. Simultaneously, the epithelium of the genital ridges proliferates and penetrates the intermediate mesoderm to form the primitive sex cords. These structures form the indifferent gonad, which begins development into the testes or ovaries at around 32–46 days of gestation in dogs [19]. Testis differentiation is triggered by the Y chromosome sex-determining region (SRY) gene, with male reproductive health largely established during the early stages of testis development. However, SRY is only transiently activated in precursor somatic cells of the seminiferous tubules (Sertoli cells), initiating their differentiation [20]. At later stages, differentiated Sertoli cells are crucial for male sex determination with primordial germ cells, testicular interstitial steroidogenic Leydig cells, and the establishment of vascular patterns. Chorionic gonadotropin controls Leydig cell formation from mesenchymal cells [21]. The fetal Leydig cell population (and additional perinatal cell line in men) is later replaced by the adult line. In the presence of androgens (produced by the Leydig cells), the mesonephric ducts (Wolffian ducts) develop to form the primary male genital ducts. They give rise to the efferent ductules, epididymis, *vas deferens*, and accessory reproductive glands. At the same time, the paramesonephric (Müllerian ducts) undergo regression in the presence of anti-Müllerian hormone, which is secreted by Sertoli cells [22]. The developmental remnant of these ducts is the appendix testis, a non-functional tissue fragment located on the upper pole of each testis. Before puberty, the activation of the HPG axis, characterized by pulsatile gonadoliberin (GnRH) secretion and the subsequent release of luteinizing hormone (LH) and follicle-stimulating hormone (FSH), facilitates the development of the adult Leydig cell population. Notably, fetal and adult Leydig cell lineages secrete androgens that differ in both quality and quantity. These hormones are responsible for the structure and function of the male reproductive system [23]. Fully developed Leydig cells of both populations stop dividing once they reach their final number and become functional; see more information in Table 1. At this stage, they produce insulin-like protein 3 (INSL3) independently of LH [22]. In most mammals, INSL3 levels remain stable (0.8–5 ng/mL) and only decrease gradually with age, reflecting the functional health or number of these cells. However, dogs are a notable exception, as they exhibit much lower INSL3 concentrations (0.02–0.46 ng/mL) [24]. Sertoli cells proliferate under FSH influence until puberty [25]. The formation of the intracellular junctions (composed of specific membrane proteins) is responsible for cell adhesion, communication, and the formation of the blood–testis barrier between adjacent Sertoli cells. This stops further proliferation of these cells. Overall, the available Sertoli cell number determines the future number of spermatozoa produced. Sertoli cells are called mother cells as they play key roles in protecting spermatogenic cells, providing nutrients and physical support for spermatogenesis [26]. These cells are classified based on stages into two types: immature Sertoli cells (from fetal to prepubertal) and adult Sertoli cells (from pubertal to post-pubertal) [27]. Of note, concurrent with hormonal changes, the testicular histology undergoes profound reorganization during puberty. Studies in dogs showed that prepubertal testes display seminiferous tubules with diameters under 100 μm until 20 weeks, containing mainly Sertoli cells and spermatogonia. However, the pubertal transition triggers remarkable structural changes in tubular diameter, increasing notably between 22 and 28 weeks to approximately 180 μm [28].

The blood–testis barrier creates a unique microenvironment for spermatogenesis. It is a dynamically remodeling structure that allows for germ cell movement and selective molecule passage. The barrier manages communication between the interstitium and the seminiferous tubules, inhibiting the development of an autoimmune response even against several cytokines and growth factors secreted by Leydig cells for intercellular signaling. It is worth highlighting here that the last findings in humans as well as in farm, accompanying animals, or wildlife indicate that endocrine-disrupting chemicals may be responsible for alterations of the blood–testis barrier that affect fertility [12,29].

The external genital, the penis, develops from the ambisexual genital tubercle, an embryonic appendage within the perineum that has some features in common with limb appendages. The main development processes involve distal outgrowth of primordia, mesenchymal condensation, differentiation of skeletal elements (*os penis*), formation of erectile bodies, polarity (dorsal–ventral, proximal–distal, and medial–lateral), muscle differentiation (smooth muscle in the penis), and epithelial differentiation [30]. Penile development comprises three layers: ectoderm forming the penile and preputial epidermis, endoderm forming most of the urethra, and mesoderm forming the erectile bodies, dermis, and connective tissue stroma. The urethral meatus constitutes an interface between ectoderm and endoderm. A particularly critical aspect of external genitalia development is androgen control that determines whether the embryonic genital tubercle differentiates into a penis or female structures.

Canine organogenesis is complete before the fetal stage begins (around 35 days post-fertilization). A cord (gubernaculum) formed from mesenchyme connects the gonad with the ventrolateral abdominal wall. It is crucial for testis descent to the scrotum in dogs at 6–16 weeks. This process is regulated by sex hormones and INSL3 [31].

## 3. Internal Reproductive Organs: Anatomy, Histology, and Hormonal Regulation

### 3.1. Testis, Spermatogenesis, and Steroidogenesis

In male dogs, the reproductive system (*organa genitalia masculina*) consists of a series of interconnected organs. The specific functional effectiveness of each reproductive structure depends on its proper anatomy and characteristic histology; for more details see Table 1. The testis is a paired endocrine gland, the primary male reproductive organ [24]. Fully mature testes are responsible for both spermatogenesis and the production of sex hormones: androgens and small levels of estrogens [32]; for more details, see Table 2 and Table 3. The spermatogenesis activation is under neuroendocrine control with a special role of GnRH and kisspeptin-releasing neurons. Kisspeptin integrates reproductive signals and metabolic status, which are essential for initiating puberty. Stimulated by GnRH, pituitary gonadotropes release gonadotropins. While the testis is divided into tubular and intertubular compartments, FSH binds to specific receptors on the membrane of Sertoli cells, which support spermatogenesis, and LH binds to receptors on the membrane of Leydig cells, regulating steroidogenesis [33,34]. The axis is controlled by negative feedback *via* testosterone from Leydig cells and inhibin from Sertoli cells. Any disturbances that occur in the activity of the HPG axis, e.g., improper stimulation or a lack of gonadotropin stimulation or hormonal feedback from the gonads, lead to fertility problems with atrophy of the reproductive organs, which is also known in dogs as canine hypogonadism [35].

**Table 1:** Anatomical, histological, and physiological characteristics of the canine male reproductive organs.

Organ/Segment	Anatomy and Histology	Cellular Details/ Layers	Physiological Role	Refs.
Testis	Covered by *tunica vaginalis* and *tunica albuginea*; contains convoluted seminiferous tubules, straight tubules (rete testis), divided into lobules.	Spermatogenic epithelium: spermatogenic cells (spermatogonia types A/Intermidiate (Int.)/B, spermatocytes, and spermatids) and Sertoli cells. The basal seminiferous tubule compartment: spermatogonia and leptotene and zygotene spermatocytes; the luminal compartment: pachytene spermatocytes, spermatids, and spermatozoa (location, morphological features, e.g., size, nuclear shape, chromatin pattern allow for the distinction of spermatogenic cells). Sertoli cells–long columnar cells with prominent triangular/round nuclei (visible closer to the basement membrane of the seminiferous tubule, on the level of spermatogonia/spermatocytes). The connective tissue between adjacent tubules: Leydig cells with a round nucleus, numerous mitochondria, smooth endoplasmic reticulum, and lipid droplets. In dogs, located in clusters near blood and lymphatic vessels) and macrophages, mast cells, fibroblasts, and telocytes. Parenchyma with peritubular-myoid cells surrounds seminiferous tubules.	Site of spermatogenesis (mitotic, meiotic, and spermiogenesis phases); primary source of sex hormones (androgens, estrogens).	[26,32,36,37,38,39]
Epididymis	Efferent ducts and subsequent ducts emerging from the testis.	Epididymal epithelium: principal cells (tall columnar with stereocilia, heterochromatic nucleus, and metabolically active lysosomes and pinocytic vesicles secreting enzymes and glycoproteins), basal cells, narrow cells, and clear cells (large endocytic cells involved in protein uptake).	Storage of mature sperm; acquisition of progressive motility (ATP-dependent) and capacitation.	[40,41,42,43]
Epididymis (Initial Segment)	Connection point to the *efferent ductules.*	Exclusively located narrow cells.	Absorption of testicular fluid; concentration of spermatozoa, luminal acidification (proton secretion for acidification).	[42,44,45]
Epididymis (*Caput*)	Head region of the epididymis.	Principal cells with long stereocilia, narrow cells, and highly active clear cells.	Fluid absorption; initiation of sperm maturation and luminal recycling.	[45,46]
Epididymis (*Corpus*)	Body region of the epididymis (less convoluted tubules)	Epithelium shorter with abundant lipids in the supranuclear region of principal cells: secretion of glycosidases and other enzymes.	Acquisition of progressive motility; biochemical changes *via* epididymosomes (attachment of proteins, e.g., AR, ER).	[42,43,46]
Epididymis (*Cauda*)	Tail–primary storage site (tubules with the widest lumen).	Thinner and shorter epithelium with prevalent clear cells.	Final sperm maturation; storage of mature sperm, detachment of cytoplasmic droplet (Hermes body).	[42,43,47,48]
*Vas Deferens* (*Ductus Deferens*)	A small, muscular transport tube.	Layers: *tunica mucosa*, *tunica muscularis* (inner circular/outer longitudinal), and *adventitial serosa* (loose connective tissue with vessels and nerves). In dogs, muscular layers consist of an inner circular layer and an outer longitudinal layer. The *lamina propria*, located beneath the *mucosa*, is a narrow connective tissue layer devoid of glandular structures and supported by dense elastic fibers. Prominent longitudinal folds; initially lined with pseudostratified epithelium with short stereocilia, transformed into simple columnar epithelium towards the distal part.	Sperm transport to the ejaculatory duct; maintenance of DNA integrity; propulsion *via* tunica muscularis.	[49,50,51,52]
Prostate	Only one accessory gland in dogs.	Oval, bilobed gland. Glandular epithelium: lined with cuboidal and columnar epithelial cells. Stroma: surrounded by a dense fibromuscular stroma with primary/secondary folds.	Secretes prostate fluid that contributes to seminal plasma (sperm survival and motility); contains high levels of DHT (dihydrotestosterone).	[53,54]
Scrotal Sac	Holds testis, epididymis, and spermatic cord; contains an evagination of the peritoneum.	*Tunica vaginalis* and spermatic fascia (derived from the abdominal wall). The *tunica vaginalis* and fascia enclose the descending testes and the spermatic cord, forming a double-layered extension of the peritoneum (inguinal canal allowing the testis to move from the abdomen to the scrotum).	Encloses and protects the descending testes and spermatic cord as a double-layered extension.	[55]
Penis	The copulatory organ. Penis glandular bulb is a specialized vascular structure of the canine penis.	The penis *tunica albuginea* contains collagen fibers and muscle fibers. Structures branching from the *tunica albuginea* extend deep into the penis, forming the walls of irregular muscular spaces (caverns: the *corpus cavernosum* and the *corpus spongiosum*) lined with endothelial cells. In dogs, the *corpus carvenosum* is of the vascular type and extends along the entire length of the penis. Highly vascularized erectile tissue.	Transferring semen to the female tract and expelling urine; erectile function is regulated by hormonal, neurovascular, and endothelial processes. Swells rapidly after copulation begins to facilitate the copulatory lock.	[55,56,57,58]

In dogs, eight stages of the spermatogenic cycle were characterized based on the acrosome system [59]; see also Table 2. Significant differences were found for the frequencies of the different stages characterized (except stages V, VI, and VIII), particularly for the Mongrel. Stage IV (spermiation) was the most frequent in different breeds, whereas stages II and VIII were the least frequent. Each spermatogenic cycle and the total duration of spermatogenesis lasted approximately 13 and 61 days, respectively, for the Mongrel, Poodle, Pinscher, Beagle, and Labrador Retriever; for the American Pit Bull, approximately 12 and 56 days, respectively [40]. Mature spermatozoa are characterized by progressive motility (midpiece mitochondria generating adenosine triphosphate, ATP). Their proper function is indicated by a stable mitochondrial membrane potential, capacitation (hyperactivated motility in the female reproductive tract), and fertilization (triggered acrosome release of enzymes that penetrate the *zona pellucida* of the egg) [60]. As spermatogenesis is a complex process with control points at every step, many errors may occur [61]. For example, non-disjunction in meiosis I results in the production of two altered cells from four, while non-disjunction in meiosis II leads to alterations in every four cells. The offspring aneuploidy is often linked to the increased parental age. The proper differentiation of spermatogenic cells relies heavily on Sertoli cell support, particularly through the production of nutrient-energy substrates, e.g., lactates and pyruvates, required at each step of spermatogenesis [41]. Moreover, Sertoli cells also provide mechanical support for spermatogenic cells as they differentiate and move towards the seminiferous tubule lumen. The blood–testis barrier divides the seminiferous tubules into two compartments: basal and luminal [62], (Table 1). Produced by Sertoli cells, sex hormone-binding globulin (SHBG) in dogs is different from that in men [46,63]; ssee more information in Table 1. In addition, gonadotropins and androgens, testicular estrogens first produced by Sertoli cells in prepubertal males and then by Leydig cells in adults (10–50 pg/mL in mammals), are also involved in sustaining spermatogenesis [64]. The testicular source of estrogen and the regulatory role of these hormones were confirmed in the 90s of the 20th century by discoveries in experimental studies in transgenic mice with knockout of genes for ERα and ERβ and P450arom, as well as clinical studies in men with mutations in the above genes, while still no intensive studies on estrogens are undertaken in male dogs [65]. Physical stress, such as heat, that affects the undescended testis primarily destroys spermatogenic cells, resulting in serious alterations of Sertoli cells and further pathological courses [66]. Studies by Luaces et al. [59] reported that heat stress (extremely high temperatures during the summer months) alters the oxidative status of male outdoor dogs, leading to an increased production of oxidative catabolites (reactive oxygen species) and reduced antioxidant enzyme activity in spermatogenic cells. This results in decreased reproductive performance due to alterations in spermatozoa. In addition, heat stress or overstimulation by LH may lead to Leydig cell hyperplasia [67]. It is worth noting that biosynthesis of sex steroid hormones by Leydig cells is a multi-level, controlled process [61]. It requires the coordinated expression of several genes and proteins with various functions (Table 3). Moreover, for cellular steroidogenic function, global lipid homeostasis is crucial. The substrate for sex hormone synthesis is stored in cytoplasmic lipid droplets and released, converted to cholesteryl esters, and transported for further conversion to mitochondria. The first-synthesized hormone, pregnenolone, diffuses across the mitochondrial membrane and is further metabolized by enzymes associated with the smooth endoplasmic reticulum by the hydroxysteroid and/or ketosteroid pathway (both active in dogs). Leydig cells express all of the enzymes essential for the conversion of cholesterol to androgens and their metabolites (including the most potent, DHT) and estrogens [68].

In dogs, androgen levels vary significantly by age or weight. At postnatal day 2, serum testosterone concentrations in male puppies are already detectable (approximately 0.15 ng/mL). In prepubertal males (16–20 weeks of age), testosterone levels remain low (0.25–0.30 ng/mL), but a significant increase occurs around 22 weeks, with levels exceeding 0.50 ng/mL, indicating the onset of puberty. In adult Beagle dogs, plasma concentrations of testosterone range between 0.70 and 1.25 ng/mL. However, considerable variation in adult non-castrated dogs (0.10–6.30 ng/mL) was also reported [28].

**Table 2:** Stages of spermatogenesis and cellular characteristics of spermatogenic cells.

Phase	Stage/Cell Type	Key Morphological and Functional Features	Duration/Marked Signs	Refs
Mitotic (Spermatogoniogenesis)	Spermatogonia (Type A, divides into stem cell/differentiating cell/Intermediate, Type B)	Rapid proliferation (mitosis) controlled by retinoic acid. Contact with the basement membrane decreases as differentiation progresses.	~60 days (Total)	[37,38,39,40,41]
Meiotic (Spermatocytogenesis)	Primary and Secondary Spermatocytes	Meiosis I and II; formation of haploid spermatids. Primary spermatocytes undergo a long prophase (~20.9 days).	Prophase (~22 days)	[34,38,41]
Spermiogenesis	Spermatids to Mature Spermatozoa	Nuclear chromatin condensation (histone to protamine transition); acrosome formation, fusion of vesicles from the Golgi complex; development of a temporary microtubular structure—manchette; tail formation from the centrioles, including the midpiece with spirally arranged mitochondria.	Species- specific	[26,41,63,69]
Spermiation	Mature Sperm Cells	Phagocytosis of excess cytoplasm by Sertoli cells; release into the seminiferous lumen.	Progressive motility (ATP- driven)	[40,41]

**Table 3:** Key enzymes and molecular regulators of steroidogenesis in Leydig cells.

Enzyme/Protein	Function in Steroidogenesis	Key Regulators	Refs
Translocator protein, and steroidogenic acute regulatory protein (TSPO and StAR)	Cholesterol release from lipid droplets and transport to mitochondria and through mitochondria membranes.	LH, cyclic adenosine monophosphate (cAMP), protein kinase A (PKA), extracellular-regulated kinase 1/2 (ERK1/2), and mitogen-activated protein kinase phosphatase 1 (MKP1).	[69,70]
P450 side chain cleavage (P450scc)	Conversion of cholesterol to pregnenolone (Initial step).	Located in the inner mitochondrial membrane.	[68,71]
3β-hydroxysteroid dehydrogenase/Δ5-Δ4-isomerase, and 17β-hydroxysteroid dehydrogenase (3β-HSD and (17β-HSD)	Conversion of pregnenolone or progesterone to dehydroepiandrosterone (DHEA) or androstenedione, respectively, pregnenolone or dehydroxypregnenolone, and DHEA, to progesterone, or 17α-hydroxyprogesterone, and androstenedione, respectively.	Smooth Endoplasmic Reticulum	[68,71]
5α-reductase (5α-Red)	Conversion of testosterone to DHT.	Crucial for the regulation of the epididymis and prostate.	[66,72]
Aromatase (P450arom)	Aromatization of androgens into estrogens.	Essential for maintaining spermatogenesis.	[59,66,72]

### 3.2. Sperm Storage and Tract Organs

The epididymis is a single, long, and highly convoluted tubule where spermatozoa are stored and finish their maturation. The rete testis tubules transform into the epididymis efferent ductules, of which the number decreases towards the *vas deferens* [44]. The lumen at the beginning is smaller in size, while at the end of the epididymis, it is wider with spermatozoa visible inside; for more details, see Table 1. In dogs, transit of spermatozoa through the epididymis takes 10–14 days [67]. Testosterone and DHT regulation is crucial for the epididymis metabolic functions, secretory, absorptive activities, and expression of essential proteins [72]. The presence of tight junctions of the blood–epididymis barrier and specific cellular transporters (epididymosomes) can regulate the composition of the epididymal cells and epididymal fluid to favor proper sperm maturation [43,46]. Of note, released during inflammation, cytokines affect barrier function, contributing to post-testicular male infertility. In addition, the epididymis is a target for therapeutic and environmental chemicals that alter spermatozoa morphology and/or functional parameters [45].

The ductus deferens (*vas deferens*) not only transports spermatozoa, but it also contributes to sperm maturation and survival (Table 1) [49,50]. The content of the ductal fluid is crucial to protect spermatozoon DNA integrity [51]. Then, spermatozoa are expelled to the short ductus ejaculatory. It was shown that vasectomy in dogs also affects the epididymis and prostate functions [53].

### 3.3. Accessory Gland: Prostate

Accessory glands produce secretions that are released into the *vas deferens* and the ejaculatory duct, thus forming the liquid portion of semen (a large portion of the seminal plasma) [68]. In dogs, only the prostate (*glandula prostatica*) is present. Like other accessory glands typical of other mammalian species, prostate function is maintained by androgens. Its anatomical location and its function are dependent on age and are closely associated with local hormonal regulation of the reproductive system [73], (Table 1).

Initially, the gland is located entirely within the abdominal cavity and maintains this position until approximately two months of age. Next, the prostate, located in the pelvic cavity until sexual maturity, enlarges in volume with the onset of maturity and expands cranially, gradually assuming a more abdominal position around 8–12 years of age [54,73]. Prostate fluid optimizes the luminal environment, thereby enhancing sperm motility and ensuring the viability of spermatozoa. It is clear, watery, and rich in chloride ions (pH 6.15–6.5 due to the presence of prostatic acid phosphatase) and represents the third fraction of the canine ejaculate. In prostate inflammation (prostatitis), pH can significantly increase (approximately to pH 7.7) [74].

## 4. Anatomy and Histology of the External Reproductive Organs

The scrotum is a muscular, skin-covered pouch divided into two parts by a central septum [53], (Table 1). The thin, hairless skin with apocrine sweat glands of the scrotum serves as a temperature regulator for the testis and cauda epididymis. The thin, hairless skin with apocrine sweat glands of the scrotum serves as a temperature regulator for the testis and cauda epididymis [71]. For the contractility of the scrotum, the dartos layer, composed of fibroelastic tissue and smooth muscle, is responsible. Thermoregulatory function is carried out in conjunction with the cremaster muscle [75]. It regulates the position of the testes by positioning the scrotum closer and further away from the abdominal wall, and the pampiniform plexus, formed by the testicular arteries and veins, which maintains cooled venous blood in maximum contact with warmer arterial blood [76].

The canine penis (*os penis*/baculum) is a characteristic anatomic organ (Table 1) with erectile function initiated by sexual arousal, molecularly regulated by nitric oxide and phosphodiesterase [77,78]. Sometimes, during copulation, swelling becomes firmly lodged behind the female vulvar muscles, leading to canine *coitus interruptus*. In this phase, the penis bends approximately 180° as the couple attempts to separate for several minutes. This anatomical positioning causes compression of the penis efferent veins, inhibiting venous return. It is confirmed that such a specific mechanism contributes to the efficient delivery of the first sperm-bearing ejaculate fraction to the uterus [78].

## 5. Genetic and Chromosomal Disorders

Domestic dog populations have been shaped by centuries of intensive artificial selection and closed pedigree practices (e.g., linebreeding, inbreeding) [55,56]. Epidemiological data reveal that reproductive system pathologies are not randomly distributed throughout the entire dog population, but rather show a significant predisposition in specific genetic lines and breeds (purebreds) [79,80]; for more details, see Table 4.

### 5.1. Sex Chromosome Aneuploidy

Canine sex chromosome aneuploidy (79, XXY karyotype) is increasingly recognized as a primary genetic cause of congenital infertility and testicular dysgenesis in dogs [77,82]. The defect is due to a meiotic non-disjunction error in gametogenesis, which is most commonly of paternal origin. This leads to an egg being fertilized by an aneuploid sperm and then creating a hyperdiploid chromosomal constitution, disrupting the functional molecular cascade of gonadal differentiation [79,95]. Clinically, although phenotypic males with distinct external genitalia may be present, the pathognomonic finding demonstrated during the andrological examination is severe bilateral testicular hypoplasia with palpably firm, fibrotic, and significantly reduced gonads relative to somatic growth [82,96]. On microscopic examination of the testicular parenchyma, a deep disruption of spermatogenesis (the seminiferous tubule sclerosis and basement membrane thickening, hyalinization) has been found [92]. Germinal aplasia (Sertoli cell-only syndrome) is a common histological finding. In response to the absence of inhibin-B feedback from dysgenic Sertoli cells and sub-optimal testosterone production. The interstitial tissue undergoes compensatory hypertrophy with diffuse or nodular Leydig cell hyperplasia. In many cases, it tends to aggregate into pseudo-adenomatous clusters that mimic neoplasia [88]. This dysregulated cellular milieu leads to a hypergonadotropic hypogonadal state, where chronically high plasma LH and FSH levels further predispose the retained dysgenetic gonads to neoplastic transformation [85]. As a result, the therapeutic consensus is that medical management with hormonal supplementation is generally contraindicated or ineffective. Early elective bilateral orchiectomy is the gold standard intervention [97]. Surgical removal is imperative not only to prevent the high incidence of Sertoli cell tumors and seminomas but also to mitigate the risks of androgen-dependent prostatic pathologies (benign prostatic hyperplasia and prostatitis) [82].

### 5.2. Male Pseudohermaphroditism

In male pseudohermaphroditism (78, XY, SRY), testes are present, but the external genitals appear feminine. In several cases, the dog has vestigial oviducts and a uterus. The testes may be located within the abdomen, the scrotum, or lateral to the vulva [98]. A penis can be present or, more often, it is an enlarged clitoris. If the penis and testes are present, the diagnosis is more difficult, and abdominal surgery is necessary to find vestigial female organs. Moreover, true hermaphroditism can be diagnosed [83]. In these animals, there are both gonadal tissues, but the secondary sex characteristics and external genitalia of the opposite sex. A testis may be found on one side, and an ovary on the other side, including the presence of an ovotestis or bilateral ovotestes. Surgical resection of the genital tract (testes, Müllerian structures) is the treatment of choice to avoid clinical problems (e.g., Sertoli cell tumors and Leydig cell tumors) and the development of neoplastic diseases associated with this disorder [99]. Male pseudohermaphroditism has been observed more often in some breeds than others (Table 4). Recent studies reported a normal male chromosome complement and a lack of the SRY and SRY-box transcription factor 9 (SOX9) gene mutations in this disease, but a mutation in the anti-Müllerian hormone type-2 receptor [100]. Karyotyping is an important test to be performed before other examinations to avoid misdiagnosis, as chronic presence often predisposes the affected tissues to secondary complications, including neoplastic transformations [99].

### 5.3. Cryptorchidism

Cryptorchidism is the failure of one or both testes to descend into the scrotum. It is considered an autosomal recessive trait and, in some cases, hereditary with tumor transformation potential [101]. It was found that, most commonly, cryptorchidism is manifested unilaterally, and the right testis stops descending more frequently than the left one [102]. If cryptorchidism affects only one testis, it may be accompanied by further tumor development in the other normal testis. Abdominal cryptorchidism predisposes to the development of Sertoli cell tumors due to a higher temperature than in the scrotum, which affects spermatogenic cells. In inguinal cryptorchidism, the temperature is higher than that in the scrotal sac but lower than that in the abdominal cavity, which may predispose to the development of seminomas. On the contrary, it was reported that higher temperature exerts no significant effect on the development of Leydig cell tumors [103]. Hernández-Jardón et al. showed that, in cryptorchid dogs, testosterone concentrations were reduced, while estradiol, LH, and FSH did not present a significant difference [104].

It is recommended to remove cryptorchid dogs from breeding programs (Table 4). The standard and recommended treatment for canine cryptorchidism is surgical removal (castration) of both testes independently of whether the second is cryptorchid or not. This allows for the prevention of testicular cancer, torsion, and the heritable trait. The results obtained by Zdunczyk et al. [105] indicated that weekly injectable testosterone therapy over 3 months was safe and capable of increasing testosterone levels in castrated dogs to within the normal range for intact dogs. In histopathological analysis of canine cryptorchid testis, high immunoexpression of desmin, inhibin α, and anti-Müllerian hormone was found [106]. Recently, it was reported that genes, e.g., *High Mobility Group AT-hook 2*; *HMGA2* (controls development) and variants in *Lysine Acetyltransferase 6A*, *KAT6A* (regulator of β-catenin, affecting histone acetylation), *RXFP2*, *INSL3* (relaxin family signaling), and steroidogenesis-controlled genes are altered, pointing to complex polygenic inheritance and epigenetic influences in cryptorchidism etiology [55]. Moreover, the last studies by Squillaciot et al. [101], revealed DNA polymorphism in the KAT6A gene, associated with changes in global histone 3 acetylation in lysine position (H3K9) and the DNA methylation pattern in the INSL3 gene, suggesting further intensive epigenetic modification research with special attention on the developmental anomalies (epididymal–testicular junction) and dog size as associated with cryptorchidism occurrence. Studies by Papazoglou et al. [107] showed decreased expression of cfa-miR-148a and cfa-miR-497 in cryptorchid testes, and the decreased expression of cfa-miR-1841 in epididymis. These molecules may be used as potential new targets for diagnosis and therapy in affected dogs. Interestingly, the last results confirmed that canine spermatogonial stem cells can be successfully used to resume spermatogenesis [108] and dogs with congenital cryptorchidism can adequately serve as a study model for human cryptorchidism [104].

## 6. Congenital and Developmental Anomalies

### 6.1. Segmental Aplasia

Segmental aplasia, a congenital anomaly, is characterized by the absence of a portion of the epididymis (typically the cauda or corpus) and/or the *vas deferens*. This defect arises from the incomplete differentiation of the Wolffian ducts, including apoptotic alterations and impaired epithelial signaling essential for tubular proliferation [94]. While the majority of clinical cases are unilateral, bilateral manifestations may also occur. Diagnostic findings through clinical examination and ultrasonography typically reveal a testis of physiological size, whereas the pathognomonic feature is the loss of continuity within the epididymal structure [84]. While the caput epididymis is usually present, the corpus or cauda terminates abruptly and/or is replaced by fibrous connective tissue. Near this blind-ended duct, cystic dilations (spermatoceles) and hard nodules, the result of sperm accumulation, are palpable [85]. The dog can still be fertile, but sperm concentration is often compromised during unilateral cases. In bilateral aplasia, the testes have active spermatogenesis, but the ejaculate does not contain sperm (obstructive azoospermia). Granulomas due to accumulated sperm at the blind terminus are the most distinct macroscopic lesion. Intraluminal pressure within the epididymal tubules increases, leading to rupture of the ductal epithelium, which in turn causes sperm to be extravasated into interstitial tissues. In response to this, the immune system induces a vigorous infiltration of macrophages, lymphocytes, and multinucleated giant cells. The inflammation process disrupts the blood–testis barrier, which leads to the production of anti-sperm antibodies, causing an irreversible sterility [88]. Although dogs with unilateral aplasia may retain reproductive capability, breeding is contraindicated due to the hereditary nature of the anomaly. In cases where sperm granulomas are infected or are distressing, bilateral orchidectomy is the safest and most effective treatment option [94].

### 6.2. Hypospadias

Hypospadias in dogs is a rare congenital anomaly. It is characterized by the termination of the external urethral orifice (meatus) at a point proximal to the penile tip (glans) along the ventral aspect, rather than at its normal distal extremity. This defect results from the incomplete fusion of the urogenital folds during embryonic development [109]. For more details; see Table 4. The known etiology involves disturbed cellular differentiation during fetal development: insufficiency in androgen masculinization signals or a lack of sensitivity in tissue receptors that prevents the midline fusion of the ectodermal cells of the urethral groove. This failure arrests the completion of the urethral tubular structure [102]. Classification of hypospadias is determined by the anatomical location of the urethral opening: glandular, penile, scrotal, perineal, or anal. In the majority of cases, a failure of ventral preputial closure is observed, presenting as a slit-like appearance in the prepuce, often accompanied by penile atrophy or an abnormal curvature known as chordee [99]. The aberrant positioning of the meatus results in an inability to direct the urinary stream, causing urine to soil the limbs or ventral abdomen. This chronic exposure causes severe urine scald and contact dermatitis. Moreover, in more severe (proximal) forms, the associated penile deformation renders coitus physically impossible [108,110]. The shortened and widened urethral path allows bacteria to ascend higher up into the bladder (cystitis risk) [106]. Asymptomatic mild (glandular) cases may not require intervention. The primary therapeutic goals are the creation of a functional urethra and the correction of cosmetic appearance (urethroplasty) [111]. In cases of severe perineal hypospadias, the most rational option is often penile amputation combined with a permanent scrotal urethrostomy (creating a urinary opening at the level of the scrotum) [85]. Studies in male dogs with pseudohermaphroditism (78, XY, SRY) identified polymorphisms not segregated with the intersexual phenotype, suggesting no link between hypospadias and polymorphism in the coding sequence of the SRY and other genes [112].

### 6.3. Phimosis

Phimosis is the inability to extrude the penis from the preputial orifice [108]. The etiology is predominantly rooted in congenital strictures. Developmental stenosis of the preputial opening mechanically prevents penile extrusion [85]. It may also present in an acquired form secondary to cicatricial tissue (scar) formation following trauma or chronic infection (balanoposthitis) [104]. Paraphimosis is characterized by the inability to retract the penis back into the preputial cavity (retraction failure), which constitutes a true urological emergency. It typically arises following coitus or masturbation, resulting from the strangulation of the penis by a constricting preputial orifice or the formation of a hair ring around the penile shaft [108]. In dogs with phimosis, the primary complication is chronic balanoposthitis due to urine pooling inside the preputial cavity. Systemic effects are rare, but the chief deficit is an inability to mate. Conversely, the clinical picture of paraphimosis is dramatic. The exposed glans penis desiccates, develops fissures, and becomes edematous, and rapidly turns cyanotic [85]. The pathophysiology of paraphimosis is a cycle of ischemic necrosis. The preputial ring constricts and initially impedes venous return of the penile blood, resulting in severe congestion and interstitial edema. At the cellular level, increased hydrostatic pressure ultimately leads to an obstruction of arterial flow, hypoxia, cellular energy depletion, and ultimately, gangrenous necrosis [107]. In phimosis, only two general changes occur inside the cell: epithelial hyperplasia and fibrosis secondary to chronic irritation. Preputioplasty (surgery for congenital strictures) aims at healing through widening of the preputial orifice. Therapeutic measures, including V-plasty or a dorsal incision of the preputial orifice, are traditionally used to allow free extrusion of the penis. The primary objective of emergency treatment is to reduce edema (*via* the application of a hypertonic solution) to the penis, which allows fluid to be removed from the tissue through osmotic pressure, and then lubricated, and then manual reduction is attempted [85]. At later stages where medical reduction has failed, or there is tissue necrosis (gangrene), phallectomy (penile amputation) is an unavoidable requirement [108,109].

### 6.4. Uterus (Uterus Masculinus)

In male dogs, a *uterus masculinus* was quite well described due to its often existing with systemic infection [69] and recently reported ovarian and ovotesticular tumors, which can be successfully diagnosed with the use of abdominal ultrasonography and computed tomography [113]. Disruption of the anti-Müllerian hormone signaling pathway or its receptor activity can enable this embryonic remnant to remain in the form of a cranial blind sac or tubular structure (paramesonephros) [82]; for more details, see Table 4. This structure is located on the craniodorsal surface or within the parenchyma of the prostate gland. In the current literature, its relationship to the urethra is considered the primary criterion for classification. In this context, thin-walled, fluid-filled structures that show anatomical communication with the prostatic urethra at the level of the *colliculus seminalis* are defined as “true *uterus masculinus*”. In contrast, forms that have no connection to the urethra, are located within the dorsal parenchyma of the prostate, are lined with glandular epithelium, and appear as a closed cavity, are described as “cystic *uterus masculinus*” [45,69,86,106]. The presence of *uterus masculinus* often results in irregular prostatic enlargement or pseudo-hypertrophy, producing false results on rectal palpation [114]. In some cases, due to hyperestrogenism, Sertoli cell tumor comorbidities take place. This metaplastic transformation results in thickening of the cyst wall, intraluminal increase in keratin, and a higher propensity for secondary bacterial infection, with potential transformation of the lesion into a massive abscess [109]. The macroscopic sequelae of the pathology are directly proportional to the volume achieved by the cyst and are the result of mechanical compression of neighboring structures. Dorsal compression of the descending colon leads to tenesmus with ribbon-like stools, and ventral pressure on the urethra precipitates dysuria or complete urinary retention, with leakage of cyst contents into the urethra. This leads to persistent hematuria and pyuria [107]. Therapeutic management is guided by the size of the lesion as well as the clinical symptom severity, including concurrent hormonal imbalances. Antibiotics or anti-androgen management is widely considered to be inadequate for the resolution of cystic structures. The gold standard of care will chiefly involve bilateral orchiectomy to remove the hormone that drives the pathologic process: epithelial atrophy, especially estrogen-dependent squamous metaplasia [106]. With larger cysts, abscesses, or due to symptoms, castration alone does not necessarily work [115]. Therefore, surgical drainage and resection are required.

### 6.5. Epididymal Cyst (Spermatocele)

An epididymal cyst (spermatocele) can occur as a separate condition. It is a fluid-filled sac in the epididymis, usually caused by sperm duct blockage, trauma, or inflammation. This pathology is common in older intact males [52]. The structure is typically small and non-painful. However, due to the potential blood–testis barrier disruption and anti-sperm antibody production, a unilateral orchiectomy can be performed as an attempt to protect the future sperm production of the remaining testicle [81]. Subsequently, the collection of semen is successfully cryopreserved for future breeding.

## 7. Age-Related, Degenerative, and Neoplastic Pathologies

In aging males, a decrease in sperm production and reproductive hormone concentrations is a reflection of changes in reproductive organ histology and function. The seminiferous epithelium vacuolization and a decrease in the number of seminiferous tubules are observed [36]. No ageing changes are detected in the seminiferous epithelium height. Collagen content in the testicular interstitium gradually increases in older dogs. Thus, there is an association between morpho-histological ageing signs: the extent of testicular fibrosis and senescence (halted cell cycle). Molecularly, age-dependent variations were detected in the sperm proteome composition related to important metabolite pathways. Therefore, several proteins need to be studied as potential aging biomarkers [114]. Aging is a risk factor for cancer development. In senior dogs (after the age of 10), the risk of cancer increases significantly. However, the lifetime risk of cancer, as well as cancer mortality, in dogs is known to vary significantly by breed. Due to new potential risk factors that are still being identified, starting cancer screening for all dogs is recommended at the age of seven, and as early as age four for breeds with a lower median age [116,117].

Current insight into male reproductive system tumor or cancer etiology is complex and links genetic predispositions and environmental influences [118]. Environmental stress impacts endocrine systems, causing imbalances in levels of sex steroids. Also, as found in working dogs, heat and chemical stressors affect sensory performance and behavior, leading to elevated levels of stress hormones and suppressed immune responses that additionally affect reproductive system function [119]. Of note, nowadays, anthropomorphic studies in dogs have found that environmental stressors, both physical and chemical, also markedly influence the psychological equilibrium and adaptive behavioral responses through stress- and anxiety-based responses [88]. It is well known that exposure to endocrine-disrupting chemicals, including phytoestrogens, induces precocious reproductive aging, including infertility as well as metabolic issues (e.g., obesity, hyperglycemia), and cancers [103,120,121]. These substances mimic or block sex hormone receptor binding and alter cell signaling, leading to uncontrolled cellular processes, gene mutations, and epigenetic modifications (e.g., DNA methylation, histone acetylation, deregulation of miRNA) [113,122]. According to studies by Harder et al. [103], nowadays, in the pathogenesis of canine testicular tumors and neoplasms, further deep research is urgently needed to explore the role of other risk factors, such as diet, physical activity, and testicular trauma.

Although detailed clinical data are still unavailable, one of the first findings in the field of canine testicular tumors on a large canine population clearly showed that incidences of testicular tumors in dogs of various ages have increased during the past decades as a result of environmental pollution [79]. For instance, testicular germ cell tumors that originate from altered primordial germ cells with an arrested differentiation cycle, modifications in chromatin, altered DNA methylation, or regulatory miRNA expression. These effects may also be exerted by environmental chemicals [123,124]. More incidences of reproductive system diseases found in male dogs may be linked, as described in humans, to the influence of both genetic and environmental factors resulting in testicular dysgenesis syndrome [89]. It includes cryptorchidism, hypospadias, and low semen parameters and tumors (Sertoli cell tumors and Leydig cell tumors, seminoma, etc., or both mixed tumors) [93,117]. In addition, it was noted that testicular tumors are mainly diagnosed in geriatric non-castrated dogs [92]. The vast majority of canine testicular tumors are benign, but malignancy potential varies among breeds or individuals [125]. It has to be highlighted that in dogs, compared to other mammalian species, a higher incidence of testicular tumors occurs [126]. The most common are seminoma, Sertoli cell tumor, and Leydig cell tumor [117,127]. At the preliminary phase of development, testicular tumors are most frequently manifested by painless enlargement of the organ, asymmetry, or a small nodule [128]. Pain develops in cases of rapid growth, bleeding to the tumor, or upon cryptorchidism. Also, azoospermia and/or oligospermia, together with dermatoses, are frequently present. Some tumor types may be hormonally active (Sertoli cell tumors and seminomas). Moreover, these multiple tumors in one testis can be identified [104,129]. The most recurrent combination is seminoma and interstitial tumors, followed by Sertoli cell and Leydig cell tumors, and seminoma and Sertoli cell tumors. The presence of three tumors within a single testicle is also observed. Very rare double tumors are found within both testes, while malignant features in mixed tumors are not rare. Evaluation of malignancy grade in canine testicular tumors is based on analysis of morphology manifested by the primary tumor (draining lymph nodes, presence of distant metastases). The first grade encompasses tumors restricted to the testes, the second grade includes metastases restricted to retroperitoneal lymph nodes and to the diaphragm, while the third grade includes distant metastases. Seminomas spread mainly by lymphatic vessels, and the metastases are located most frequently in retroperitoneal lymph nodes. Non-seminomas yield metastases both through lymphatic and blood vessels, mainly to the lungs, liver, bones, and brain. Malignant forms of Sertoli cell tumor and Leydig cell tumor develop metastases mainly to the inguinal and sublumbar lymph nodes [103,129].

### 7.1. Testicular Tumors

#### 7.1.1. Germ Cell Tumor

Seminomas mainly affect older dogs. The typical age at diagnosis of testicular tumors in dogs is approximately 10 years old [91,116,117]. The neoplastic germ cells are large and distinct, with a vesicular nucleus (one or two nucleoli) and scant cytoplasm. They appear macroscopically as solid, whitish, gray, or pale-yellow tumors with diameters ranging from 0.5 to 3 cm [129]. Cryptorchidism is one of the leading risk factors of seminomas [91,117]. Seminomas are typically non-active, but rare studies report hormone-producing seminomas causing feminization. Seminomas may be soft, homogeneous, gray or cream, and well-circumscribed on the macroscopic evaluation. According to the World Health Organization (WHO), seminomas are classified into two primary histological patterns: the intratubular pattern, which is generally benign, and the diffuse pattern, which is associated with a higher potential for malignancy [128]. In the intratubular form, neoplastic cells remain confined within the seminiferous tubules, while in the diffuse tumor, cells breach the basement membrane and infiltrate the interstitial tissue, where they aggregate into extensive cellular layers [117]. Furthermore, like in human medicine, the classification of canine seminomas can be narrowed into classical seminoma (originating from primordial germ cells) and spermatocytic seminoma (affecting aged dogs 10–12 years old) [122]. A comprehensive list of biomarkers used in the diagnosis of seminoma is presented in Table 5.

The primary treatment option is bilateral orchiectomy (castration), which is usually curative in cases without metastasis [117]. Seminomas are quite radiosensitive compared to other testicular tumors. Therefore, radiotherapy is an effective treatment in cases suspected of regional lymph node metastasis [131]. In advanced metastatic cases, chemotherapy protocols based on cisplatin or carboplatin are recommended [125]. It is reported to be a classic type or spermatocytic, common type, and usually benign [128]; for more details, see Table 4. It is often found in older, intact males, especially with cryptorchidism. Findings show that the expression level of miR-302c-3p is decreased in testes with seminoma [107]. Castration provides a high cure rate. However, it can cause feminization or bone marrow issues. Spada et al. [132] reported the first successful use of radiation therapy with a short-period protocol in a Yorkshire terrier with abdominal seminoma and persistent Müllerian duct syndrome. 

#### 7.1.2. Sertoli Cell Tumor

Sertoli cell tumors are more common in younger dogs [116]. The main clinical finding of Sertoli cell tumors is palpable enlargement in the affected testis and atrophy in the contralateral testis due to negative feedback from paraneoplastic hyperestrogenism [133]. Most tumors are benign but show the potential to metastasize [134]. Sertoli cell tumors are considered to be characterized by feminization syndrome because of excessive estrogen. Hyperestrogenism can lead to severe disease states, including bone marrow hypoplasia/bi-hypoplasia. A Sertoli cell tumor may be associated with spermatic cord torsion [117]. Histologically, neoplastic Sertoli cells can be columnar and have distinct cell borders. These tumors show intratubular growth and consist of elongated cells in single or double lining in the seminiferous tubule (cell nuclei are occasionally present at the core or the base of the cell) [128]. A comprehensive list of biomarkers used in the diagnosis of Sertoli cell tumors is presented in Table 6.

The preferred treatment is bilateral orchiectomy. In cases without metastasis, surgical intervention is usually curative and leads to regression of feminization symptoms within weeks [133,135]. To prevent recurrence and tumor development in the other testis, bilateral castration is recommended [133].

#### 7.1.3. Leydig Cell Tumor

Leydig cell tumors are one of the three most common tumor types in non-castrated male dogs and have been reported to account for 23% to 58% of tumors in various studies, particularly in younger dogs [117,126]. However, some reports show that over 60% of Leydig cell tumors are diagnosed in older dogs [136]. Metastasis of this tumor was also reported to the lungs and bones [117,135]. These tumors are least associated with cryptorchidism [17]. However, if such feminization signs (mammary enlargement, estrogenic alopecia, penile shrinkage) are present, they can be halted by castration [137]. In cases of bilateral Leydig cell tumors, estradiol levels were increased, LH decreased, and FSH remained normal. Conversely, in dogs with unilateral tumors, peripheral blood analysis revealed increased estradiol concentrations alongside low testosterone levels, although there are also reports of elevated testosterone in some instances [127]. It is postulated that aromatase activity may be potentiated by a tumor-derived factor [134]. In the tumor mass, normal Leydig cells and/or cells with scarce endoplasmic reticulum, polymorphous nuclei, and cristae-type mitochondria are present [138]. Macroscopically, Leydig cell tumors are well-circumscribed lesions with a homogeneous yellow appearance on the cut surfaces, ranging from golden brown to light brown. They can show hemorrhage and/or cysts [129] and distinct focal hypoechoic lesions. During B-flow, color, and power Doppler studies, Leydig cell tumor vascularization is perilesional or mixed morphology, while in other tumors, intralesional vascularization prevails [139]. A comprehensive list of biomarkers used in the diagnosis of Leydig cell tumors is presented in Table 7.

Bilateral orchiectomy is advocated as the treatment of choice and is curative because of its very small metastatic potential. Moreover, perineal hernias require castration to reduce the chances of recurrence [135]. Our recent study in search of new biomarkers of this tumor with the involvement of next-generation sequences revealed, for the first time, the complete transcriptome of canine Leydig cell tumor [140]. These were, e.g., upregulated expression (982 transcripts) and downregulated expression (168 transcripts). With the use of KEGG and Gene Ontology enrichment analyses, we found that a significant proportion of differentially expressed genes are directly involved in the control of sex steroid production, *CYP11A1*, *STAR*, and *3β-HSD3B1*, or tube formation, angiogenesis, and extracellular matrix remodeling in interstitial cells; *ESM1*, *FGG*, and *VEGFA*. Moreover, upregulated expression of transcripts responsible for neurotransmitter or neuroendocrine signaling: *SLC6A4*, *GRIN2C*, *GBRB3*, and cholesterol metabolism and its regulation, *GPX3*, *MSMO1*, *DHCR24*, were revealed. The upregulated genes were associated with the kinase PI3K-Akt cascade and extracellular matrix interactions. These are a common feature of various tumors, including human Leydig cell tumors, and are currently a well-developed clinical tool. However, determined here also, changes in estrogen signaling and relaxin signaling seem to be distinctive, not well-studied mechanisms in the canine Leydig cell tumor. A large number of genes downregulated in canine Leydig cell tumors encoded proteins implicated in development including angiogenesis (*DMRTC2*, *SEMA3C*), cellular process including differentiation (*DMC1*, *NCAPH*, PEG10), cell migration, adhesion (*EPCAM*, *FAT1*), immunoregulatory mechanisms (*ALOX12*, *ALOX5*, *CKLF*), cell signaling including via neurotransmitters (*ASB16*, *CHGA*, *SPATA46*) with implication to the above *MIR8905*, *PRAME PNMA6F* which are still under investigation. All the mentioned transcripts, when their expression is altered, were confirmed to be highly contributing to tumorigenesis mechanisms [135].

### 7.2. Prostate Disorders

Pathologies occurring in the prostate are grouped into four main categories: benign prostatic hyperplasia, prostatitis, prostatic cysts, and prostatic neoplasia [141]. Benign prostatic hyperplasia, being one of the most prevalent diseases of the reproductive system of dogs, is an age-dependent and nearly unavoidable condition of non-castrated dogs [142]. The disease is androgen-dependent and related to changes in the estrogen/testosterone ratio. Dihydrotestosterone causes hypertrophy of stroma and glandular epithelium [89]. Although histological features begin in most dogs older than 6 years, this condition is present in over 95% of dogs reaching 9 years of age [142]. While most cases present with subclinical onset, in severe cases, there can be tenesmus (straining), ribbon-like defecation, hematuria (bloody urine), and bloody discharge from the urethra as a result of the pressure of the prostate on the rectum [141]. Bilateral castration reduces prostate volume approximately 50% in 3 weeks and 70–75% in 9 weeks following an operation. In dogs with high breeding value, a 5α-reductase inhibitor is widely used because it decreases prostate size without altering semen quality [88]. It has been demonstrated that serum prostate-specific esterase level measurement is helpful, as it reflects prostate volume in diagnosis [143]. Moreover, multiparametric ultrasonography (mpUS), which exceeds the limitations of standard ultrasonography, has revolutionized the characterization of prostate lesions [133]. Concomitantly, analysis of total testosterone is still useful in gland growth monitoring [144].

#### 7.2.1. Prostatic Neoplasia

Prostatic neoplasia (cancer), despite being a rare disease observed in dogs (0.2–0.6% prevalence), is an aggressive disease with high metastatic potential [135]. Adenocarcinoma and transitional cell carcinoma are the most common types. Interestingly, castration has no protective effect on prostate cancer, but it has been reported that cancer incidence may be higher or follow a more aggressive course in castrated dogs [145]. Ultrasonographic picture shows a very heterogeneous appearance and the presence of calcification of foci (strong suspicion for prostate cancer, particularly in castrated dogs); for more details see Table 4. Due to diagnosing the disease at an advanced disease stage (with bone and lung metastases), prognosis remains very poor as the mean term of survival after diagnosis is only 1–2 months. Recently, an unusual case of prostate cancer metastasis to the epaxial muscles and myocardium was reported [136]. Molecular analyses indicate that tumors are often poorly differentiated and possibly originated from prostatic collecting ducts, showing both prostatic epithelial and urothelial cell expression profiles (e.g., uroplakin III positivity). Furthermore, intraepithelial neoplasia lesions may be precursor lesions for adenocarcinoma [146]. In treatment applications, nonsteroidal anti-inflammatory drugs inhibiting cell proliferation and accelerate apoptosis are used. Also, Gibson et al. [147] showed that Death Domain Associated protein (DAXX), which stands for Alpha-thalassemia/mental retardation X-linked (ATRX), may be a useful biomarker for this cancer in dogs. Despite its low incidence, the aggressive nature and high metastatic potential of canine prostate cancer make it a serious clinical problem. Very little data exists on the molecular mechanisms and regulatory networks underlying prostate neoplasia development. Transcriptional analysis reveals a markedly reduced expression of the voltage-dependent calcium channel alpha2delta1 subunit in canine prostate cancer compared to benign prostatic hyperplasia, which can be a promising target for diagnosis and therapy [148].

#### 7.2.2. Prostatic Cyst

Differences in etiology exist in cystic pathologies affecting the canine prostate gland. Intraprostatic cysts generally originate in hyperplastic glands in older dogs from secretory stasis or urine reflux *via* urethral fistulas. In contrast, paraprostatic cysts arise from embryonic Wolffian duct remnants adjacent to the prostate. Intraprostatic cysts have a heterogeneous clinical presentation correlating to pathology. Small lesions usually remain subclinical until masses are produced by prostatic enlargement. Often, these cysts are diagnosed with benign prostatic hyperplasia or prostatitis. In the advanced stages, clinical signs may include abdominal distension, pain, and general malaise. Moreover, severe complications like cyst rupture or secondary bacterial infection may produce an acute abdomen and sepsis [141]. The diagnostic evaluation in dogs is mainly performed by radiographic and ultrasonographic imaging. The cysts on ultrasound are usually thin-walled, spherical lesions with an anechoic or hypoechoic echotexture. Sometimes internal sediment or septations are present. Additionally, ultrasonography permits fine-needle aspiration, enabling the collected fluid to be investigated for potential cytological and bacteriological analysis [125].

#### 7.2.3. Prostatitis

Prostatitis, inflammation of the prostate gland, usually arises when an ascending bacterial infection from the urethra invades prostatic tissue that is frequently already complicated by benign prostatic hyperplasia or cysts [141]. It occurs in both intact dogs and castrated dogs [148]. Acute prostatitis is characterized by systemic manifestations, such as fever, depression, and severe abdominal pain, whereas chronic prostatitis presents with more insidious symptoms, potentially only recurring urinary infections or decreased semen quality. Abscesses are cavities of purulent fluid that develop secondary to chronic prostatitis, and rupture can result in life-threatening complications, including peritonitis or septicemia. *Escherichia coli* is the most common isolated microorganism, although *Mycoplasma canis*, *Staphylococcus* spp., *Streptococcus* spp., *Pseudomonas* spp., and *Brucella canis* are also responsible for the infections. Infection, especially with *Brucella*, must be evaluated. Furthermore, superinfections can happen [1]. Diagnostic testing depends almost purely on ultrasonography and bacteria population in the culture of prostatic fluid. Treatment consists of high-lipid solubility antibiotics that permeate the blood–prostate barrier for at least 4–6 weeks, and follow a course of castration to eliminate the underlying biologic predisposing factors [149].

## 8. Infectious Diseases

### 8.1. Infectious Sexual Tumors

Canine transmissible venereal tumor is a clonally derived neoplasm that involves canids. Its unique transmissible mode is by physical allogenic transfer of living cancer cells during coitus. Histopathologically, this tumor is classified as a mesenchymal round cell tumor [137]. Macroscopically, the lesions present as solitary or multiple masses on the *mucosa* of the prepuce, penis, vagina, or vulva. Such growths are usually referred to as nodular, papillary, or having a distinct cauliflower-like appearance [117]. At the cellular level, neoplastic cells are seen as large, round, or oval, containing fine granular chromatin, a prominent nucleolus, and cytoplasmic punctate vacuoles. Macroscopic spread and immunity evasion from cellular sources is endemic and appears to be especially pronounced in free-roaming dog populations, with constant, uncontrolled mating. It ranges widely across geographic areas, such as Eastern Europe, and globally [137]. Testicular tumors, especially when they reach large sizes, can lose tissue integrity, forming widespread necrosis and bleeding foci. This can lead to ulceration of the tumor or hemorrhagic discharge from the prepuce [133]. The one most important cell-level effect of a tumor is probably its immune avoidance mechanism. In the developing stage, tumor cells suppress major histocompatibility complex (MHC) class I and II molecule expression, which can prevent the host cells from producing a cytotoxic T-cell response [137]. Although the rate of metastasis is between 5% and 17%, the tumor is mainly local and can also spread to regional lymph nodes [117,137]. Surgical therapy is considered inadequate for this disease because of anatomical limitations and high recurrence rates (30–70%), so chemotherapy is currently the most effective treatment choice. The most frequently used protocol is to provide intravenous vincristine sulfate. Reduction of tumor volume and a cytological decrease in neoplastic cell vacuolization are monitored for therapeutic response [117].

### 8.2. Balanoposthitis

Balanoposthitis is characterized by concurrent inflammation of the penile (balanitis) and preputial (posthitis) *mucosa*, commonly encountered in intact male dogs [150]. A chronic and untreated condition can be linked to penile cancer. The etiology tends to be a transformation of commensal microflora in the preputial cavity, for example, *E. coli* and *Streptococcus* spp. into opportunistic pathogens, trauma, and foreign bodies are also predisposing factors [145]. The most characteristic clinical presentation is a yellow-green purulent or mucopurulent discharge, which leaks from the preputial orifice. It is necessary to distinguish this pathological discharge from the physiological generation of whitish, hypocellular smegma [139,145]. In cases of chronic balanoposthitis, the preputial *mucosa* appears hyperemic and thickened, exhibiting a characteristic ‘cobblestone’ nodular appearance due to lymphoid follicle hyperplasia [150]. Healthy dogs typically show few squamous epithelial cells and extracellular bacteria on their swab samples. On the other hand, cytology in cases of balanoposthitis reveals significant degenerate neutrophil infiltration and active phagocytosis [139]. The inflammatory process causes a breakdown of mucosal integrity, resulting in local irritation, pain, and persistent licking, causing a mucosal injury that is prone to secondary trauma [150]. In severe, untreated cases, edema and fibrosis may develop, narrowing the preputial orifice (phimosis), preventing penile extrusion and consequently, copulation [88,89]. The pathogenesis is driven by the disruption of local immune homeostasis within the preputial cavity and the subsequent overgrowth of aerobic bacterial populations, leading to microbial dysbiosis [145]. Mild-to-moderate cases generally respond to conservative therapy. Aggressive and unnecessary systemic antibiotic use should be avoided to prevent the emergence of resistant strains. The gold standard of treatment is regular lavage of the preputial cavity with antiseptic solutions (e.g., diluted chlorhexidine or povidone-iodine). This mechanically removes organic debris and reduces bacterial load [150,151]. For intractable, recurrent, or severe follicular forms, the use of specific systemic antibiotics based strictly on culture and sensitivity results is indicated.

## 9. Conclusions

A complete understanding of the physiological functions, regulations, as well as pathological processes and diseases of the canine reproductive system is crucial for the design of research and correct diagnosis and management. Therefore, current experimental and clinical andrology must be based upon the integrated transfer of data information on developmental, genetic, aging, and environmental factors that directly or partially contribute to the physiopathology of the reproductive system. Moreover, analyzing the literature from human clinical data is important for following and understanding guidelines, as well as for careful translation and prediction of answers regarding the state of the individual organ and organism. Only such an approach allows us to manage the reproductive health of our companion animals in the face of new challenges and threats.

## 10. Future Perspectives

In light of the knowledge presented here, canine andrology still requires dynamic and cutting edge technologies in clinical studies and research, especially in: (i) advanced diagnostic tool development (e.g., early, fast genotyping, molecular markers, ultrasonography), (ii) advanced in vitro techniques to study the molecular basis, particularly of developmental processes, together with reproductive biotechnology methods (e.g., creation of canine primordial germ cells culture, spermatogenic stem cell culture, immortalized cell lines, and infertility treatment including assisted fertilization), (iii) improvements of breed control and contraception techniques (e.g., *via* use of safe and successful hormonal/enzyme blockage for reversible sterilization or modified action of specific enzymes, and optimizing semen cryoconservation), and (iv) standardization and integration of clinical practices and knowledge (with special attention on used pharmacological agents, existing and new external physical and chemical factors, and their effect on canine fertility and induction of reproductive system diseases).

It is important to add that, although advanced diagnostic approaches are available nowadays, they are still not often used for the evaluation of the reproductive system. Multiparametric ultrasonography (mpUS) allows for the timely analysis of both biomechanical and functional properties of tissues, surpassing the distribution of standard B-mode ultrasonography [3,4]. Elastography (Shear Wave—SWE and Strain—SE), contrast-enhanced ultrasonography (CEUS), and Doppler-based techniques provide this structural complementary information. Elastography contributes to lesion characterization by providing both qualitative and quantitative (m/s or kPa) assessment of tissue stiffness; healthy testicular tissue typically shows low-to-moderate elasticity values (approximately 1.40–1.65 m/s), while tumors show significantly higher performance values at the level of malignant, purulent orchitis, and fibrotic changes. With the use of microbubble contrast agents, CEUS allows for a detailed explanation of microvascular perfusion. In addition, quantitative perfusion clearance, such as peak density and mean transit time, was known to be attainable through semen quality and sperm motility. Color, power, and pulsed wave Doppler techniques allow for the quantitative evaluation of testicular blood analyses (Peak Systolic Velocity, End Diastolic Velocity, Resistive Index/Pourcelot Index, Pulsatility Index/Gosling Index). The occurrence of intratesticular and supratesticular arterial growth velocities can be accurately found in spermatogenic dysfunction and infertility.

Another important point that needs to be strongly emphasized once again is that our accompanying animals are chronically exposed to environmental chemicals and also toxins. Household dust, furniture, plastics, and commercial pet products, phthalates, bisphenols, and several persistent organic pollutants such as polychlorinated biphenyls and polycyclic aromatic hydrocarbons can be absorbed through inhalation, ingestion, or skin contact. They exert a wide range of negative effects, including disturbances in reproductive function as well as thyroid function, neurological processes (anxiety, depression), immunological processes (dermatoses), cardiovascular processes, metabolic processes (diabetes, obesity), and adrenal dysfunctions (Addison’s disease). There is a clear need for further studies across all the aforementioned areas. 

## Figures and Tables

**Table 4:** Breed-specific predispositions in canine reproductive pathologies.

Disease/Anomaly Type	Predisposed Dog Breeds
Chromosomal anomalies	Jack Russell Terrier and mixed-breed dog [81], Maltese, Miniature Schnauzer [82],
Male pseudohermaphroditism	Doberman Pinscher, Pug, Cocker Spaniels, French Bulldogs, Miniature Schnauzers (more often than in other breeds), Mixed-breed dog (Canino Mestizo), American Cocker Spaniel, Bichon Frise [77,78,83].
Cryptorchidism	English Bulldog, Boxer, Chihuahua, Shetland Sheepdog, Siberian Husky, Yorkshire Terrier, Cairn Terrier, Chihuahua, Miniature Dachshund, Maltese, Pekingese, Pomeranian, Toy, Miniature and Standard Poodle, Miniature Schnauzer, Old English Sheepdog, Shetland Sheepdog, German Shepherd, Maltese Dog and mixed-breed dog [57,58,82].
Hypospadias	Boston Terriers, Dalmatian [84].
*Uterus masculinus*	Miniature Schnauzers, German Shepherd, Yorkshire Terrier, Maltese and mixed-breed dog [85,86,87,88]
Seminoma	Boxers, Weimaraners, German Shepherd, Golden Retriever [89,90,91].
Leydig cell tumor	German Shepherd, Crossbreed dogs, and mixed- breed dog [81,82,83,84,85,86,87,88,89,90,91,92].
Various testicular tumors	Yorkshire Terrier, Labrador Retriever, Fox Terrier [93].
Prostate carcinoma	German Shepherd, Rottweiler, American Staffordshire Terrier, Scottish Terrier, Berger de Beauce, and Bernese Mountain Dog [94].

**Table 5:** Biomarkers used for immunohistochemical diagnostic differentiation and prognosis of canine seminomas.

Biomarkers/Stainings	Description
c-KIT proto-oncogene receptor tyrosine kinase (c-KIT; CD117)	Classic seminoma (spermatocytic seminomas are usually negative) [91,128].
Placental alkaline phosphatase (PLAP)	Classic seminomas (spermatocytic seminomas are negative) [91,128].
Periodic acid-Schiff staining (PAS)	Classic seminomas [128].
-Sal-like protein 4 (SALL4) -Protein gene product 9.5 (PGP 9.5)	Used for the identification of germ cells in different stages [111].
-Double sex- and mab-3-related transcription factor (DMRT1) -Deleted in azoospermia& (DAZ)	Originating from spermatogonia [15].
Ki-67	Proliferation index and positivity [91,128].
Cyclin-dependent kinase inhibitor 2A (p16)	Mediated aging [130].
Estrogen-related receptors (ERRs)	Potential new markers [18,63].
-Periostin -Podoplanin	Malignancy analysis via phosphatidylinositol 3-kinase (PI3K)/protein kinase B (Akt) signaling and cell migration processes [117,125].

**Table 6:** Biomarkers used for the immunohistochemical diagnosis of canine Sertoli cell tumor.

Cell Tumor Type	Positive Biomarkers	Description
Sertoli Cell Tumor	Cytokeratin (CK, AE1/AE3), Vimentin, Inhibin-A, Ani-Müllerian hormone (AMH)	Cytoskeletal markers and Sertoli cell secreted hormones are used as the gold standard in Seminoma differentiation. Frequent cytokeratin expression supporting partial epithelial differentiation of neoplastic Sertoli cells [127,128].
	-Periostin -Podoplanin	Kinase PI3K-Akt signaling and cell migration processes [125]
		Sertoli cell tumors do not express c-KIT (CD117) protein [91,128].

**Table 7:** Biomarkers used for the definitive diagnosis of canine Leydig cell tumors.

Cell Tumor Type	Positive Biomarkers	Description
Leydig Cell Tumor	Inhibin-A, Melan-A, Calretinin, Vimentin, c-KIT (CD117), Ki-67.	Malignant Leydig cell tumors exhibit increased Ki-67 expression compared to benign ones [91,128].

## Data Availability

No new data were created or analyzed in this study.
